# Comparative evaluation of serum zinc level in head and neck cancer patients before and after radiation therapy

**DOI:** 10.22088/cjim.14.1.128

**Published:** 2023

**Authors:** Danial Fazilatpanah, Hamid Fallah Tafti, Sara Rasta, Masume Masudian, Atefe Rangani

**Affiliations:** 1 Cancer Research Center, Babol University of Medical Sciences, Babol, Iran; 2 Babol university of Medical Sciences, Babol, Iran; 3 Non-communicable Diseases Research Center, Alborz University of Medical Sciences, Karaj, Iran; 4 Semnan University of Medical Sciences, Semnan, Iran; 5 General practitioner, Babol, Iran

**Keywords:** Serum zinc, Head and neck cancer, Radiation therapy

## Abstract

**Background:**

Head and neck cancers (HNCs) include 5.3% of all cancers and they are the cause of the same 5.3% of cancer deaths. Oxidative stress has a crucial role in cancer progression and cancer therapy. Ionizing radiation causes cell malfunction and death by creating reactive oxygen species. Due to its antioxidant activity, immune system power enhancement and role in apoptosis, zinc is a crucial trace element in oncology including HNCs. We decided to compare serum zinc level of HNC patients before and after RT, to assess the potential effects of ionizing radiation therapy on serum zinc.

**Methods:**

Fifty-seven HNC patients, who were candidates for curative radiation therapy (RT), were enrolled and their serum zinc level just before and 2 months after completion of RT were checked in a single laboratory. RT was prescribed by linear accelerator with 60 to 70 Gy by conventional method. Data were analyzed by SPSS 20.

**Results:**

Mean serum zinc prior to RT and following RT were 77.64±13.45 mg/dl and 68.28±11.93 mg/dl, respectively, which was lower following RT (p<0.001). Patients’ sex, age and duration from diagnosis to treatment and site of disease didn’t have any impact on serum zinc difference.

**Conclusion:**

This study showed that RT of HNCs leads to serum zinc reduction, which is greater in nodal disease because of either larger field or higher dose of radiation. Taking zinc supplements while being treated by RT, may be necessary.

Head and neck cancers (HNCs) generally refer to cancers of upper aero-digestive tract including lips, paranasal sinuses, nasal cavity, nasopharynx, oral cavity, hypopharynx, cervical esophagus and also thyroid gland (1). In 2017 about 890,000 new cases of HNC were diagnosed representing almost 5.3% of all cancers. Reportedly, they were causes of the same 5.3% of deaths due to cancer (2). Tobacco, alcohol consumption and human papillomavirus (HPV) are three main etiologic factors of these cancers (3). Overall, a mixture of external and internal factors initiates cellular neoplasia which gradually progresses into carcinoma. Oxidative stress, a physiologic state in which high levels of reactive oxygen species (ROS) and free radicals are generated, plays a crucial role in both cancer progression and cancer therapy (4-6). In terms of cancer treatment, some means of cancer therapy, specifically radiotherapy (RT) do their role by creating ROSs in tumor cells; which subsequently lead to cancer cell malfunction and death (7-9). Zinc is an essential trace element especially in the field of oncology which could be dangerous both in low and high levels (10, 11).

Zinc has an anti-oxidant activity as a component of superoxide dismutase enzyme, which catalyzes ROSs thus preventing generation of other toxic free radicals (10, 12-15). Zinc has many more functions in oncology including its role in enhancing immune system power, acting as antagonist of iron and copper- two oxidant factors, stabilizing biological membrane and initiation or` inhibition of apoptosis- the main mechanism of cell death (10, 16).

There have been many reports which show the relation between zinc deficiency and occurrence and prognosis of some HNCs. Hashemian et al. analyzed Golestan cohort study regarding the role of zinc deficiency on incidence of esophageal squamous cell carcinoma and suggested a significant negative correlation (17). Joshaghani et al. by comparing serum zinc level of high and low incidence regions regarding esophageal cancer, found significant lower serum zinc level in high risk regions of Golestan province, Iran (18).

Among HNCs a recent study by Lubinski et al. concluded that higher zinc levels increase survival of laryngeal cancer patients (19). Prasad et al. observed lower disease-free survival, larger tumor size and more advanced overall stage of HNC patients in their review (20). Regarding the role of radiation therapy Mahdavi et al. concluded that by performing RT, mean daily energy and protein intake of HNC patients is in direct relation with mean zinc level significantly (21). Given the unclear role of RT of HNC on serum zinc level specially in Iran, the aim of this study is to compare serum zinc level of HNC patients before and after course of RT.

## Methods

In this cross-sectional study, 57 adult patients with histologically approved HNCs who were candidates of adjuvant RT in Emam Reza hospital, Mashhad, Iran were included. Ethics approval letter, was taken from ethical committee in biological researches of Mashhad Islamic Azad University. Patients with either history of previous RT or history of taking zinc supplement within last two months were excluded. Written informed consent was obtained from all patients. Demographic data including age at the time of RT, sex, duration from diagnosis to treatment were collected. In addition, latest American Joint Committee of Cancer staging for each patient was recorded. One cc blood sample were obtained from each patient prior to RT and samples were submitted to Emam Reza hospital laboratory. Then the patients were irradiated by linear accelerator with 60 to 70 Gy, 2 Gy/Fr in 6 to 7 weeks; from Saturday to Wednesday, 5 days per week. Two months after completion of RT, again, one cc blood sample were obtained from each patient. Zinc level of all samples were checked by PerkinElmer (Norwalk, CT, USA) Zeeman 3030 spectrometer in a single laboratory by flame method. Finally, the collected data were analyzed by SPSS 20. First, the data were assessed by Kolmogorov–Smirnov test and then if results were normal, T-test was used; otherwise, Wilcoxon signed-rank test was used. Level of statistical significance for p-value was considered <0.05.

## Results

This study was conducted on 57 patients with HNC, undergone RT at Emam Reza hospital, Iran. Minimum age, Maximum age and mean age of patients were 24, 90 and 57.7 years, respectively. There were 38 (66.7%) male and 19 (33.3%) female patients. Regarding time from diagnosis to treatment; minimum, maximum and mean duration were 3, 60 and 16 months, respectively. 70% of patients had local disease, 26.7% had regional (nodal) disease and 3.3% had metastatic disease. In terms of primary tumor site, pharyngeal tumors including nasopharynx, oropharynx and hypopharynx were the most prevalent; which included 26.3% of patients. Table 1 shows frequency distribution regarding primary tumor site. Mean serum zinc prior to RT and following RT were 77.64±13.45 mg/dl and 68.28±11.93 mg/dl, respectively; which was significantly reduced following RT (p<0.001). Serum zinc difference among males and females were -8.95±6.61 mg/dl and -10.16±3.31 mg/dl, respectively (p>0.05).

**Table 1:** Frequency distribution of tumor site

Tumor site	Number	Percent
**Pharynx**	15	26.3
**Larynx**	7	12.3
**Oral cavity**	11	19.3
**Thyroid**	13	22.8
**Nasal and paranasal sinuses**	11	19.3
**Total**	57	100

In case of duration from diagnosis to treatment, the net difference of serum zinc was not significantly different between two groups of less or more than one year. The difference in group of less than one year was -12.26, and in group of more than one year was -8.30, with a p-value of >0.05. Regarding age groups, we divided patients into three subgroups of 20-49, 50-69 and ≥70 years old and compared the difference between serum zinc among these subgroups by Kruskal test, which is shown in table 2. There was not significant difference between age groups by comparing the one-by-one serum zinc difference between each two groups by Man-Whitney test (p>0.05 in each three subgroups).

**Table 2:** Mean serum zinc difference in age groups

Age group (year)	Mean serum zinc difference (mg/dl)	p-value
**20-49**	-9.15	0.569
**50-69**	-10.67	
**>70**	-7.97	

In case of disease stage, we compared the difference of mean serum zinc in local versus regional disease. The differences were -6.62±8.15 mg/dl and -14.20±7.73 mg/dl in local and regional disease, respectively and Man-Whitney test showed a significant difference between these subgroups. (p<0.05). Table 3 shows these data. And finally, regarding primary tumor site, although as is shown in table 4, there were no significant difference between different sites, the greatest difference was observed in thyroid cancer patients. Paired comparison of all sites together by Man-Whitney test shows insignificant difference between each two groups (p>0.05 in each subgroup).

**Table 3:** Mean serum zinc difference regarding disease stage

Disease stage	Mean serum zinc difference (mg/dl)		p-value
**Local**		-6.62	0.024
**Regional**		-14.2	

**Table 4:** Mean serum zinc difference regarding primary tumor site

Tumor site	Mean serum zinc difference (mg/dl)	p-value
**Pharynx**	-12.49	0.31
**Larynx**	-9.87	
**Oral cavity**	-9.36	
**Thyroid**	-14.28	
**Nasal and paranasal sinuses**	-1.20	

## Discussion

Zinc is an anti-oxidant trace element which stabilizes DNA, RNA and organelle structure within the cell (22). It acts as an important factor for DNA synthesis which is a crucial step for wound healing (23). It has been proven that oral zinc sulfate could prevent mucosal toxicities of ionizing radiation therapy of HNCs (24). On the other hand, RT is one of the main treatment modalities for HNCs, therefore, assessment of the effect of RT on serum zinc in HNCs would be important. We conducted this study to measure serum zinc level of 57 patients with HNCs in Mashhad, Iran, before and after RT. This study showed that serum zinc in post-RT samples is significantly lower than pre-RT samples. Neither patients’ sex, nor duration from diagnosis to treatment, affect serum zinc in this study. Likewise, we couldn’t show any significant effect of disease stage as local or nodal, on serum zinc level. Finally, regarding primary tumor location among six different sites of head and neck including pharynx, larynx, oral cavity, thyroid gland, skin and nasal and paranasal sinuses, again there was not any significant difference between subgroups; although serum zinc difference was higher among thyroid gland cancer primaries.

As mentioned earlier, the main finding of current study is that serum zinc level decreases after radiation therapy among HNC patients. There are some similar studies on serum zinc level. Gorgu el al. compared serum zinc in two groups of HNC patients and concluded that serum zinc following treatment was significantly lower than normal in the group in which zinc supplements was not prescribed for, in comparison with the group who took zinc supplements (25). Kudva et al. in a case-control survey in 2021, showed lower serum zinc in HNC patients in comparison with healthy control group (26). Lower serum zinc in cancer patients could be attributed to lower food intake, lower bioavailability, higher phytate-zinc ratio in food and higher ROSs in cancerous cells. Gumulec et al. in a meta-analysis concluded that serum zinc is lower in many cancers such as lung, HNC, stomach, liver, breast and prostate (27).

Najafizade et al. showed that zinc supplement administered by 150 mg per day during RT and until one month after RT completion, resulted in prevention and reduction of the radiation effect on sense of tastes (28). Probably taste sensation disorders due to RT is a cause of malnutrition and subsequent zinc deficiency, which can be treated by taking zinc supplements. In another study on zinc supplements in HNC, Lin et al. reported 3-year survival benefit among whom the supplement was taken (29). In a similar study, Buntzel et al. reported that serum zinc decreases from 0.76 to 0.55 mmol/l in HNC during a 17-month follow-up (30). They also reported very low serum zinc in HNC patients in 4 to 6 weeks prior to death. They concluded that serum zinc concentration may be a marker for definitive palliative situations in HNC patient

There were two main limitations in the current study which may have influence on final conclusion. First is the small sample volume and second is the lack of a control group consisting of cancer patients who were not treated by RT of head and neck region. A suggestion for future studies would be to design a case-control study with the same propose as this study. Another suggestion is to run a study to compare the effect of different doses of RT and chemotherapy regimens in HNC on serum zinc level. Another point that should be mentioned is that a relatively large percent of the patients in this study were thyroid cancer patients although the indication of curative radiotherapy for this patients are limited. This is because of performing the study in a referral center for this cancer which may influence the results.

This survey showed a significant reduction in serum zinc among HNC patients following RT. Considering that RT may cause toxicities such as malnutrition, it may be reasonable to administer zinc supplements to compensate the abovementioned deficiency, prevent subsequent complications, and improve quality of life and survival rates.
